# The Link between Attachment Style and Self-Reported Olfactory Ability: A Preliminary Investigation

**DOI:** 10.3390/brainsci11101367

**Published:** 2021-10-18

**Authors:** Amy Shell, Anna Blomkvist, Mehmet K. Mahmut

**Affiliations:** 1 Food, Flavour and Fragrance Lab, School of Psychological Sciences, Macquarie University, Sydney 2109, Australia; amy.shell@students.mq.edu.au (A.S.); mem.mahmut@mq.edu.au (M.K.M.); 2 Department of Psychology, Stockholm University, 11419 Stockholm, Sweden

**Keywords:** attachment, olfactory ability, olfaction, romantic relationships, body odors

## Abstract

Individuals in healthy romantic relationships gain significant benefits to their psychological wellbeing and physiological health. Notably, the majority of relationship research has focused on how adult attachment influences these relationship outcomes while the role of olfaction remains an emerging research focus. The aim of the current study was to bring together these seemingly unrelated factors–attachment and olfaction–in an online quasi-experimental design. The participants were 401 undergraduate students, predominantly females, ranging in age from 17 to 70 years. Participants completed a battery of questionnaires that evaluated their attachment tendencies, olfactory ability and experiences in romantic relationships. Results indicated that attachment insecurity, across both attachment anxiety and avoidance, was associated with decreased olfactory functioning for females. These findings provide preliminary evidence that olfaction is related to romantic relationship maintenance and suggests that body odors could be fundamental for evoking the attachment system. These findings also elicit enticing new avenues of research which can assist psychologists to provide targeted treatments to individuals with olfactory deficits and insecure attachment tendencies.

## 1. Introduction

A person’s olfactory ability may affect the initiation, maintenance and dissolution of a romantic relationship [1]. Another strong predictor of experiences within and outcomes of romantic relationships is adult attachment style. Despite the theoretical links between olfactory ability and adult attachment style, no previous studies have empirically explored this relation. Therefore, the aim of the present study was to investigate whether adult attachment style is associated with olfactory ability, specifically, whether an individual’s attachment style differs based on their olfactory ability. The Introduction presents a review of the attachment and olfaction literature separately, culminating in a demonstration of how adult attachment style and olfactory ability are theoretically related.

### 1.1. Attachment

Adult attachment was first conceptualized by Bowlby [2], through his study of the infant caregiver bond. Following Harlow’s experiments [3], Bowlby theorized that the bond between caregiver and child was built upon the facilitation, or lack thereof, of safety, comfort and security. Initially, physical proximity to the primary caregiver represents a secure base of safety and safe-haven for the child to return to when distressed [4]. With the secure base nearby, the child can confidently explore their world and begin trusting others, consequently influencing their mental schemas about themselves, others and the world [5]. Bowlby described this bond as the attachment system, whereby the primary caregiver becomes the child’s attachment figure they call to when distressed.

How the attachment figure reacts to the infant’s distress calls determines how the infant learns their own value; how trustworthy others are; and how others will protect, love and care for them [6]. The differences in these responses lead to several observable reactions upon reuniting with caregivers following a stressful situation. These reactions allowed Ainsworth and colleagues [7] to identify attachment styles, defined as relatively stable traits known as attachment avoidance and attachment anxiety. These traits are conceptualized as existing on continuums of attachment security, from secure to avoidant and secure to anxious. Notably, higher tendencies of attachment anxiety or attachment avoidance are also described as insecure attachment. Upon reaching adulthood, an individual will develop analogous attachment from their primary caregiver towards a romantic partner, whereby their romantic partner becomes the attachment figure and safe-haven [8].

### 1.2. How Does Attachment Relate to Romantic Relationship Initiation, Maintenance and Dissolution?

Schindler and colleagues [9] found that although individuals with anxious attachment tendencies reported a greater willingness to commit to long-term relationships, they did not report a higher frequency of actually being in committed relationships. Therefore, while individuals with anxious attachment tendencies have a greater desire to commit, they might not possess the capabilities to acquire a partner as easily. In contrast, individuals with avoidant attachment tendencies were significantly less likely to enter a committed relationship than both individuals with secure and anxious attachment tendencies [9]. Hence, greater insecure attachment tendencies can predict a reduction in romantic relationship initiation. Taken together, this may mean that individuals with higher attachment avoidance or anxiety are at a greater risk of the reduced wellbeing typical for individuals without romantic and social relationships [10,11,12].

When individuals with insecure attachment tendencies do commit to an intimate partner, they often report significantly greater relationship distress, lower relationship satisfaction and shorter relationship longevity [13,14]. Therefore, even after committing to a relationship, individuals with greater avoidant or anxious attachment still have difficulty reaping the psychological and physical wellbeing benefits of a healthy, successful romantic relationship.

As apologies pose a pivotal feature of mediation during relationship turmoil, Schumann and Orehek [15] examined the apologies used after an individual had made a transgression against their romantic partner. The researchers found that higher avoidant attachment tendencies were associated with the use of more defensive responses and fewer apologetic elements, which hindered relationship reconciliation. Similarly, in a study over ten weeks, Feeney and Noller [16] found that individuals with higher avoidant attachment tendencies were significantly more likely to report termination of their romantic relationship. These findings illustrate the tendency for individuals with higher avoidant attachment to employ self-sabotaging behaviors within romantic relationships.

In contrast, individuals with anxious attachment tendencies are more likely to exhibit pathological jealousy within their romantic relationship [17]. This can often lead to relationship dissatisfaction, conflict, violence and, ultimately, relationship termination [17]. These individuals were also more likely to engage in jealousy-inducing behaviors (e.g., flirting with others, lying about the existence of a rival partner) to punish their partner and test their commitment [18]. Jealousy-inducing behaviors also have detrimental implications for the continuation of the relationship [18], highlighting that individuals with anxious attachment tendencies may have difficulty maintaining their romantic relationships.

In summary, adult attachment can be highly influential on relationship outcomes, whereby insecure attachment tendencies, both avoidant and anxious, predict poorer relationship functioning and increased instances of relationship termination.

### 1.3. Olfaction

Prima facie, it may appear that olfaction is unrelated to relationship outcomes, yet recent literature has demonstrated that olfactory ability is highly influential on one’s social functioning. Although most perception research has been dominated by visual and auditory investigations, more recent research has built a solid foundation for understanding olfactory development in infancy to adulthood and its involvement in social and romantic relationships [1,19].

Before birth, a fetus will learn to identify odors through the mother’s diet and display preference towards these smells immediately after birth [20,21]. In early infancy, olfaction plays a significant role in the identification of kin, as both mothers and newborns utilize olfactory cues to recognize each other and show preference towards each other’s odors [20,22]. Even later in life, both mothers and fathers can identify the developmental stage of their children simply from their body odors [21,23], as well as distinguish between the body odors of their own children aged between three and eight [24]. These findings have also been replicated among non-kin, whereby individuals were capable of identifying the body odor of a friend compared to strangers’ [25,26].

An infant’s peak olfactory functioning occurs around the age of six. This was demonstrated by Chalouhi and colleagues [27], who found that healthy children, aged six years or more, displayed similar olfactory functioning to adults. However, due to this rapid development in early years, upon reaching the age of 30, one’s olfactory ability typically begins to decrease due to numerous age-related diseases and cumulative damage [28,29,30].

### 1.4. How Do Olfactory Deficits Affect Quality of Life?

Olfactory deficits are strongly linked with decreased safety, enjoyment of food and quality of life [28,31,32]. For example, not being able to smell a gas leak [33] or spoiled food [31]. Moreover, individuals who suffered from chronic nasal diseases which reduced their sense of smell reported that upon treatment of their olfactory condition, their depreciated quality of life had returned to healthy levels [34], highlighting the influence of olfactory functioning on quality of life.

Impaired olfactory ability may also negatively impact social bonding [35]. This occurs partly because body odors are an invisible, chemosensory signal for the identity, emotional state and immune-functioning of another individual [36,37]. For example, Prehn-Kristensen and colleagues [38] demonstrated that the insula, a brain region associated with feeling empathy, became activated in the presence of anxiety-related sweat, yet not for sport related sweat. This suggests that individuals unconsciously communicate their stress levels through sweat, precipitating the necessity of an empathetic response from bystanders.

Odors are also effective at triggering vivid emotions and memories [39]. This is partly due to the location of the olfactory bulb which is linked via neural networks to the amygdala and hippocampus, both brain regions associated with emotions and memories [39]. Granqvist and colleagues [40] demonstrated the effectiveness of olfactory signals at influencing emotions by administering electrical shocks to induce stress in participants. The participants reported a significant reduction in the experience of discomfort when smelling their romantic partner’s body odor compared to a pleasant rose smelling chemical, especially so for the more securely attached individuals. This research illustrated that a critical feature of olfaction in social functioning is replicating physical closeness, such as to one’s attachment figure, which is possible due to the strong association between emotions, memories and olfaction.

Further evidence of the connection between olfaction and romantic relationships comes from Croy and colleagues [41] who found that congenitally anosmic women reported higher degrees of romantic relationship security, whereas congenitally anosmic men reported fewer sexual partners than those with an intact sense of smell. From these findings, Croy and colleagues [41] posited that olfactory deficits depreciate a male’s exploratory behaviors. Since the inability to rely on these chemosensory signals reduces their confidence in reading potential romantic interests within a social context, this leads to a decrease in the initiation of romantic relationships. Further evidence for the link between olfaction and close romantic relationships comes from McBurney and colleagues’ [42], who found that 87% of women and 56% of men engaged in “comfort smelling”, that is, smelling items that contain their partner’s scent when they were not present.

### 1.5. The Present Study

While the research findings presented above indicate having a secure attachment and a good sense of smell are conducive to healthy romantic relationships, no study has specifically investigated whether the attachment dimensions are linked to olfactory functioning. Therefore, the aim of the current study was to determine whether olfactory ability was related to the attachment dimensions. It was hypothesized that attachment insecurity would be associated with lower self-reported olfactory ability. In addition, we hypothesized that attachment insecurity would relate to measurement of odor awareness. In terms of the relationship survey, it was lastly hypothesized that attachment insecurity would be associated with lower relationship longevity and, lastly, attachment avoidance would be associated with fewer serious relationships.

## 2. Materials and Methods

### 2.1. Participants

Four-hundred and one undergraduate psychology students, predominately female (81.5%) and heterosexual (81.8%), completed the study for course credit. Participant ages ranged from 17 to 70 years old, and the mean age was 20.83 (*SD* = 6.91). Participants were recruited between March and June 2020 through one of two ways. The first was based on the scores on an attachment survey undergraduate psychology students completed as part of a larger screener study (described below). Specifically, we invited, via email, 99 participants who completed the attachment survey and whose scores indicated the greatest tendencies of attachment anxiety or the greatest tendencies of attachment avoidance. Of the 99 participants who were invited, 40 ultimately participated in the current study. The second method of recruitment was via an advertisement on the University’s participant recruitment website with no exclusion or inclusion criteria. The ethical aspects of this study were approved by the University Human Research Ethics Committee and all participants gave informed consent before participating.

### 2.2. Measures Screener Survey

All students taking an introductory psychology course completed a screener survey which consisted of various measures submitted by researchers to help them identify and recruit participants with specific characteristics for subsequent studies. The screener survey included the Experiences in Close Relationships–Short Scale [43], which was included to identify participants with relatively high levels of anxious and avoidant attachment due to the low incidence rate within the population [8].

#### Experiences in Close Relationships—Short Form (ECR-S)

The ECR-S [43] is a 12-item questionnaire which measures two dimensions: (a) attachment anxiety and (b) attachment avoidance, including six items for each facet. The questionnaire uses a nine-point response scale ranging from *disagree very strongly* (1) to *agree very strongly* (9). Attachment anxiety items included statements such as “I need a lot of reassurance that I am loved by my partner.” In comparison, attachment avoidance items included statements such as “I try to avoid getting too close to my partner.”

Dimension scores were calculated by summing the response options scores after reverse scoring negative items. Scores could theoretically range from 12 to 54 for both the anxiety and avoidance facets, with a higher score indicating greater attachment insecurity. Reliability analyses indicated that the ECR-S demonstrated strong internal consistency (α = 0.81 for anxiety and α = 0.91 for avoidance), while Wei and colleagues [43] reported adequate internal consistency (α = 0.77 to α = 0.86 for anxiety and α = 0.78 to α = 0.88 for avoidance), good test-retest reliability and reasonable construct validity.

### 2.3. Main Study Measures

#### 2.3.1. Demographic and Relationship History Questions

Demographic information including sex, sexuality and age was collected. Participants also reported on their experiences in romantic relationships, including their relationship status, the length of their longest relationship and the number of serious relationships they have been in.

#### 2.3.2. Experiences in Relationships Questionnaire (ECR)

The ECR [44] is the extended version of the ECR-S, presented above, and is a 36-item questionnaire that measures two facets: (a) attachment anxiety and (b) attachment avoidance, with 18 items for each. The questionnaire uses a seven-point scale with response options ranging from *strongly disagree* (1) to *strongly agree* (7). Items for the anxiety facet include statements such as “I worry about being alone” while statements for the avoidance facet include “I find it difficult to allow myself to depend on my romantic partner.”

Facet scores are calculated by reverse scoring the required 10 items and summing the item values which ranges from 18 to 126. A total score of overall attachment insecurity is created from the sum of all 36 statements, ranging from 36 to 252. Higher scores for these indicate greater attachment insecurity and lower scores indicate attachment security. The ECR is recognized to have excellent internal consistency (α = 0.91 for anxiety and α = 0.94 avoidance; 44) and good test-retest reliability [43]. Based on data from the current study, the ECR also has excellent internal consistency (α = 0.94 for attachment avoidance, α = 0.92 for attachment anxiety and α = 0.91 for attachment insecurity).

#### 2.3.3. Health Screener

Questions were utilized to assess participant’s current and previous health as relevant to their sense of smell. Participants indicated whether they had previously had any head or nose related injuries or operations, their smoking status and any medical conditions that may compromise their sense of smell.

#### 2.3.4. Odor Awareness Scale (OAS)

The OAS [45] is a 33-item scale designed to measure an individual’s general awareness of odors. Participants respond to 31 questions on a range of five-point scales, such as, *never* (1) to *always* (5) for the statement “When a room has an unpleasant smell, does it influence your mood?” Alternatively, two items used four-point scales, such as, *I will never return there* (1) to *I will not let my shopping be influenced by the way a supermarket smells* (4) for the item “Suppose you are at a supermarket where it smells bad. Is this a reason for you not to return there?”

A total score is calculated from the sum of all responses, which ranges from 32 to 163 with a higher score indicating greater awareness of odors. Analyses from the current study indicate that the OAS has excellent internal consistency (α = 0.90), while Smeets and colleagues [45] reported strong construct validity and good internal consistency for the OAS (*α* = 0.80).

#### 2.3.5. Sense of Smell Questionnaire (SSQ)

To calculate self-reported olfactory ability, participants were asked two questions: (a) How well can you smell odors? (b) How good would you consider your sense of smell compared to others? Participants answered these questions on a five-point scale ranging from *exceptionally well/well above average* (1) to *very poorly/well below average* (5). An olfactory ability score was created for participants from the sum of their responses, ranging from 2 to 10, with a higher score indicating greater self-reported olfactory ability. According to Lötsch and Hummel [46] self-reported olfactory ability, although limited, is reasonably accurate at measuring a participant’s olfactory functioning. This self-reported olfactory ability measure demonstrated good internal consistency (α = 0.80) and was correlated to the surrogate measure (*r* = 0.59), olfactory awareness, in the same manner that Smeets and colleagues [45] reported, therefore indicating that self-reported olfactory ability in the current study is a sound measure of actual olfactory functioning.

### 2.4. Procedure

Participants completed the survey online using Qualtrics in their own time, which took approximately 20 min. Following the provision of consent, participants completed the measures in the following order: the demographic and relationship history questions, health screener, Sense of Smell Questionnaire, Experiences in Close Relationships Scale and Olfactory Awareness Scale.

### 2.5. Statistical Analysis

Data was analyzed using Stata version 16.1 [47]. Spearman Rank order correlations were performed to examine the relation between olfactory measures, attachment measures and relationship variables as Shapiro Wilks indicated some variables were non-normally distributed (*p*s < 0.05). *t*-tests were also utilized to compare olfactory scores between males/females. Where necessary, non-parametric statistics were analyzed with Mann-Whitney tests. We note that one participant was significantly older (i.e., 70) than the mean age we did not exclude them from the analyses as the pattern of findings emerged with and without their data. The critical alpha level was set at 0.05 and we corrected for multiple comparisons using a Bonferroni adjustment.

An attention-check question was embedded in the middle of the ECR survey to assess participant’s understanding of instructions and continued attention of the survey requirements. The data of thirteen participants who did not answer the attention-check correctly were not included in the analyses and participants who selected “other” for their sex (*n* = 4) were not included in analyses that included sex as a binary variable. If participants selected the response option, “I choose not to answer this question” on any item, their response was recorded as a missing value.

A Mann-Whitney test revealed females had significantly higher self-reported olfactory ability scores than males (*U* = 9138, *z* = −1.99, *p* = 0.047, *r* = −0.10), so analyses were separated by sex.

## 3. Results

### Hypothesis Testing

Initially, descriptive statistics were analyzed for the dependent variables. See Table 1.

**Figure 1:**
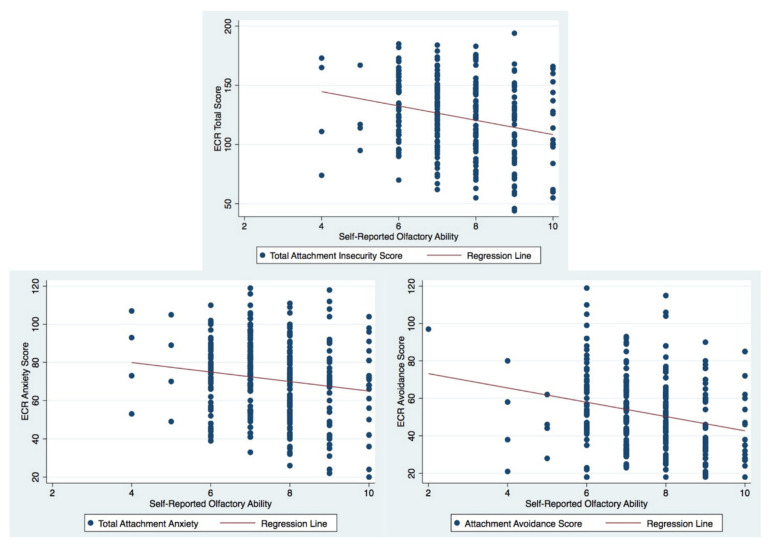
Raw data scatterplots between self-reported olfactory ability and attachment measure scores for females.

To assess the first hypothesis, Spearman rank order correlations were conducted to investigate whether the attachment dimensions were correlated with self-reported olfactory ability. Results (see Table 2) indicated that attachment insecurity was modestly, negatively correlated to self-reported olfactory ability for females only (see Figure 1). Further, a stronger relationship was evidenced between attachment avoidance and olfactory ability than attachment anxiety.

The second hypothesis was examined through Spearman rank order correlations assessing the relationship between attachment style and olfactory awareness. The results indicated a modest, negative relationship was prevalent between attachment avoidance and olfactory awareness for females. For further details see Table 2 and Figure 1 for a graphical display of the correlation between attachment measures and self-reported olfactory ability among females

To evaluate whether the relationship factors were associated with attachment styles per the final hypothesis, Spearman rank order correlations were conducted between the attachment measures and relationship variables. Results (see Table 3) indicated a similar pattern of results between males and females whereby all attachment measures were moderately negatively correlated to number of serious partners and relationship length.

In addition, further Spearman rank order correlations were conducted to examine whether the relationship factors were associated with the olfactory measures. Results indicated no significant correlations between self-reported olfactory ability and number of serious partners or relationship length. However, the results indicated a moderate, positive correlation between olfactory awareness and number of serious relationships, as well as relationship length among females (see Table 4).

## 4. Discussion

The aim of the current study was to evaluate how olfactory ability was associated to the attachment dimensions. As hypothesized, attachment insecurity, across both attachment anxiety and attachment avoidance, were modestly, negatively correlated to self-reported olfactory ability for females. In addition, the same pattern of results was found for olfactory awareness. These findings are consistent with Croy and colleagues’ [41], who found that females with no sense of smell reported significantly higher degrees of romantic relationship insecurity than females who were capable of smelling. These results suggest that greater attachment insecurity is related to reduced olfactory functioning.

One explanation for these results stems from the theory that body odors are utilized as a subconscious form of social communication [38,48]. Therefore, a reduction in olfactory functioning results in a reduced capacity to perceive and/or accurately interpret these non-verbal and non-visual forms of communication. Thus, to perceive or to have olfactory awareness could have an impact on the communication in close romantic relationships and further, actually mimic interpersonal behaviors, such as attachment avoidance and anxiety tendencies. Furthermore, our findings indicated that better olfactory awareness was associated with having longer relationships and a greater number of serious romantic partners, suggesting that perhaps being more aware of body odors can be romantically beneficial to an individual. Research has also demonstrated that olfactory deficits depreciate an individual’s quality of life through reduced social functioning [35], social security [41], and increased social anxiety [49]. Thus, similarly, the relation between reduced olfactory functioning and attachment insecurity may compromise one’s ability to successfully interpret their partner’s body odors, consequently contributing to anxiety surrounding the relationship, portrayed in their attachment insecurity.

An alternative explanation is that the reduced capability to smell a partner’s body odors depreciates an individual’s capacity to evoke the attachment system and feel reassured in times of distress, thus negatively contributing to their attachment security. In essence, smelling an attachment figure’s body odor might reproduce a similar effect of physical proximity to the attachment figure when an individual is distressed, in alignment with Granqvist and colleagues [40] research discussed previously. Although attachment is a relatively stable trait, it is not unheard of for attachment tendencies to be altered with repeated exposure to contradicting beliefs and values, such as the consistent reduction in feeling reassured from an attachment figure [50].

In terms of the sex discrepancy for these findings, the fact that these results were only demonstrated among females is consistent with Croy and colleagues’ [41], discussed previously, and could be explained because females typically portray greater empathy, emotional awareness and emotional intelligence than males [51,52,53]. Further, as odors are particularly effective at triggering vivid emotions and memories [39], females may therefore be more influenced by a reduced capacity to comprehend body odors as a means of social communication, whereas males may be less affected.

Finally, consistent with our hypothesis, we found that, among females, attachment insecurity was negatively correlated with relationship length and attachment avoidance was negatively correlated with number of serious partners. These findings are consistent with earlier studies [13,14] that also reported an association between relationship longevity and attachment insecurity among both males and females.

Similarly, consistent with Schindler and colleagues [9], who reported that attachment avoidance was associated with fewer relationship partners, among our sample, we found that attachment avoidance was related to having fewer serious relationships for females. These findings are typical of the largely prevalent research which highlights that attachment insecurity is associated with poorer romantic relationship outcomes. Moreover, these findings strengthen the proposition that females are influenced more substantially in their relationships, whether by attachment insecurity or olfactory deficits.

### 4.1. Limitations

There are three primary limitations of this study. First, although self-reported olfactory ability is a reasonable predictor of an individual’s sense of smell, it is not perfect. Prior to COVID-19 social distancing, this study included a face-to-face portion to assess olfactory ability using Sniffin’ Sticks [54], and in person ratings of body odor disgust sensitivity. As it was not possible to go ahead with this testing, future research should re-examine these results utilizing psychophysical face-to-face testing.

A second limitation was that age was strongly skewed towards a younger population. Considering olfactory functioning tends to depreciate from 30 years onwards [30], it cannot be determined whether the findings would generalize to older populations. However, when examining the relationship between total attachment insecurity and olfactory ability, controlling for age, Kendall’s partial Tau indicates that the correlation remains statistically significant, *r_τ_*(289) = −0.17, *p* < 0.001. Finally, because we only assessed olfactory ability, it remains possible that those with high attachment insecurity may show a generalized tendency to report a poorer ability in other tasks or modalities (e.g., cognitive tasks, visual perception).

### 4.2. Implications and Directions for Future Research

The relationship between attachment insecurity and reduced olfactory ability and awareness for females has wider implications for the field in understanding how body odors may play a role in the maintenance of romantic relationships, particularly within the attachment system. Notably, body odors appear more important for females than males in terms of facilitating relationship security. Therefore, as females appear to rely more on olfactory cues, olfactory deficits may be more disadvantageous for females than males in romantic relationship maintenance. Researchers should consider using olfaction as a quality of life indicator for individuals with a reduced sense of smell.

Future research should also consider examining the extent to which participants with reduced olfactory functioning report smelling their absent partner’s body odors. Moreover, future research would benefit from assessing other abilities (e.g,. cognition, visual perception) to determine whether high attachment insecurity is exclusively associated with poorer olfactory ability. These findings would assist in understanding the relationship between attachment insecurity and olfactory deficits by exploring whether the olfactory deficiency reduces an individual’s desire and capacity to evoke the attachment system and feel reassured in times of distress. Identifying this relationship might also allow for the development of treatments or targeted therapy practices for clinical psychologists and patients with olfactory deficits.

## 5. Conclusions

Overall, this study contributes to the present literature by demonstrating that relationship insecurity is associated with poorer olfactory ability. This finding highlights the significance of olfaction in social communication and emotional connection among females and raises tantalizing future research opportunities including identifying if treating olfactory dysfunctions can improve an individual’s attachment insecurity. Such research might lead to greater insights for psychologists to provide more targeted treatment practices.

## Figures and Tables

**Table 1:** Descriptive Statistics for Dependent Variables.

	Mean (SD)	Range	*n*
Attachment Anxiety (ECR)	70.87 (20.77)	99	367
Attachment Avoidance (ECR)	52.65 (20.00)	101	365
Attachment Insecurity (ECR)	123.81 (29.86)	150	359
Length of Longest Relationship (yrs.)	2.40 (2.37)	9.8	268
Number of Serious Relationships	1.27 (1.30)	6	390
Olfactory Awareness (OAS)	121.61 (17.72)	89	353
Self-Reported Olfactory Ability	7.44 (1.35)	8	388

Note. ECR = Experiences in Relationships Questionnaire; Maximum length of longest relationship participants could select was 10 years; Maximum number of serious relationships participants could select was 6; OAS = Olfactory Awareness Scale.

**Table 2:** Correlations Between Attachment and Olfactory Measures by Gender.

	Females			Males		
	Attachment Insecurity	Attachment Avoidance	Attachment Anxiety	Attachment Insecurity	Attachment Avoidance	Attachment Anxiety
Self-Reported Olfactory Ability	−0.24 **	−0.24 **	−0.15 **	−0.10	−0.02	−0.10
Olfactory Awareness	−0.09	−0.18 *	−0.05	−0.00	−0.08	−0.11

Note. Spearman’s rank-order correlations (*r*s) were performed where Shapiro Wilks indicated that some variables were non-normally distributed (*p*s < 0.05). For females, sample sizes ranged from 269 to 299. For males, sample sizes ranged from 57 to 64. * *p* < 0.05, ** *p* < 0.01.

**Table 3:** Correlations Between Attachment and Relationship Measures by Gender.

	Females			Males		
	Attachment Insecurity	Attachment Avoidance	Attachment Anxiety	Attachment Insecurity	Attachment Avoidance	Attachment Anxiety
Number of Serious Partners	−0.24 **	−0.32 **	−0.09	−0.30 *	−0.22	−0.24 *
Length of Longest Relationship	−0.36 **	−0.29 **	−0.24 **	−0.09	−0.08	−0.15

Note. Spearman’s rank-order correlations (*r*s) were performed where Shapiro Wilks indicated that some variables were non-normally distributed (*p*s < 0.05). Longest relationship length participants could select was 10; Maximum number of serious partners that participants could select was 6. For females, sample sizes ranged from 211 to 299. For males, sample sizes ranged from 44 to 63. * *p* < 0.05, ** *p* < 0.01.

**Table 4:** Correlations Between Olfaction and Relationship Measures by Gender.

	Females		Males	
	Self-Reported Olfactory Ability	Olfactory Awareness (OAS)	Self-Reported Olfactory Ability	Olfactory Awareness (OAS)
Number of Serious Partners	0.09	0.22 **	0.13	0.12
Length of Longest Relationship	0.12	0.22 **	0.02	0.12

Note. Spearman’s rank-order correlations (*r*s) were performed where Shapiro Wilks indicated that some variables were non-normally distributed (*p*s < 0.05). Longest relationship length participants could select was 10; Maximum number of serious partners that participants could select was 6. For females, sample sizes ranged from 201 to 315. For males, sample sizes ranged from 40 to 67. ** *p* < 0.01.

## Data Availability

The data is not available due to confidentiality reasons.
